# Are We Setting Doctors Up to Succeed? A Benchmarking Audit and Quality Improvement Project of Psychiatry Induction Identifying and Addressing MDT Working and Referral Confidence in Intellectual Disability Psychiatry

**DOI:** 10.1192/bjo.2026.11430

**Published:** 2026-06-30

**Authors:** Rhea Mathews, Feroz Nainar, Richard Chater, Owen Elias, Sonal Gohil

**Affiliations:** BCHC NHS Foundation Trust, Birmingham, United Kingdom

## Abstract

**Aims:**

Starting a new post can be intimidating when induction varies between trusts. This project aims to look at doctors’ experiences with induction within different NHS trusts in the West Midlands. A secondary aim was to use the survey as a benchmarking exercise, comparing experiences at Birmingham Community Healthcare (BCHC) (psychiatry trainees rotate through intellectual disability (ID) placements) compared with other trusts. Initial audit identified limited understanding of MDT working in ID Psychiatry, highlighting need for greater support at induction.

**Methods:**

An online survey was distributed to doctors who started posts in psychiatry across different NHS trusts. It had multiple choice and free text questions covering pre-start information, induction quality, IT systems and team inclusion. The standard was induction provides new trainees with timely IT access and clear understanding of MDT roles and referral confidence. Quantitative data was summarised as percentages, while free-textresponses were reviewed to identify common themes. Following identification of uncertainty around MDT working in BCHC a separate MDT confidence survey was distributed to assess trainee confidence in MDT referral process within ID service.

**Results:**

Initial survey comparing induction experiences across trusts in the West Midlands had 28 doctors respond. Clear pre-induction information reported by 61% of doctors. Overall induction rated as good by 64% with 21% rating it poor. 79% felt they had what they needed to be productive but access to key resources were delayed with 61% receiving laptop on day one, while 22% waited two weeks. Administrative issues reported by 57% of doctors in first week, 29% completed mandatory training in their time, but 93% of doctors felt welcomed. In contrast, BCHC implemented a structured induction and IT access at trust headquarters and appointments booked from the following week. The secondary survey focused on MDT confidence had nine resident doctors within BCHC complete it. 78% felt confident working within the MDT however confidence varied by referral type. 56% felt confident referring to physiotherapy compared to 44% for psychology and OT. 67% reported hesitating to refer, 56% delayed referrals and only 23% felt confident making timely referrals.

**Conclusion:**

This benchmarking audit highlights a significant variation in induction experiences across trusts with lack of efficient processes. It highlights a lack of confidence to refer to our MDT colleagues within ID Psychiatry. Based on survey findings, targeted intervention of an MDT guide and teaching session for new trainees was conducted at BCHC for February 2026 intake. Survey on MDT confidence has been undertaken by the new cohort prior to the intervention and will be repeated in 6 weeks to assess impact of the new MDT guide for trainees.

